# History of the Growing Burden of Cancer in India: From Antiquity to the 21st Century

**DOI:** 10.1200/JGO.19.00048

**Published:** 2019-08-02

**Authors:** Robert D. Smith, Mohandas K. Mallath

**Affiliations:** ^1^ King’s College London, London, United Kingdom; ^2^ Tata Medical Center, Kolkata, India

## Abstract

This review traces the growing burden of cancer in India from antiquity. We searched PubMed, Internet Archive, the British Library, and several other sources for information on cancer in Indian history. Paleopathology studies from Indus Valley Civilization sites do not reveal any malignancy. Cancer-like diseases and remedies are mentioned in the ancient Ayurveda and Siddha manuscripts from India. Cancer was rarely mentioned in the medieval literature from India. Cancer case reports from India began in the 17th century. Between 1860 and 1910, several audits and cancer case series were published by Indian Medical Service doctors across India. The landmark study by Nath and Grewal used autopsy, pathology, and clinical data between 1917 and 1932 from various medical college hospitals across India to confirm that cancer was a common cause of death in middle-aged and elderly Indians. India’s cancer burden was apparently low as a result of the short life expectancy of the natives in those times. In 1946, a national committee on health reforms recommended the creation of sufficient facilities to diagnose and manage the increasing cancer burden in all Indian states. Trends from the Mumbai population-based cancer registry revealed a four-fold increase in patients with cancer from 1964 to 2012. Depending on the epidemiologic transition level, wide interstate variation in cancer burden is found in India. We conclude that cancer has been recognized in India since antiquity. India’s current burden of a million incident cancers is the result of an epidemiologic transition, improved cancer diagnostics, and improved cancer data capture. The increase in cancer in India with wide interstate variations offers useful insights and important lessons for developing countries in managing their increasing cancer burdens.

## INTRODUCTION

Cancers are caused by mutations that may be inherited, induced by environmental factors, or result from DNA replication errors.^1^ Aging is the main risk factor for carcinogenesis in multicellular animal organisms including humans.^2-4^ Cancer is ranked as the first or second leading cause of death in 91 of 172 countries and is third or fourth in an additional 22 countries.^5,6^ Cancer is the second and fourth leading cause of adult death in urban and rural India, respectively.^7^ Cancer is now the leading cause of catastrophic health spending, distress financing, and increasing expenditure before death in India.^8-10^ Out-of-pocket expenditure (OOPE) is three times higher for private inpatient cancer care in India.^9^ Approximately 40% of cancer costs are met through borrowing, sales of assets, and contributions from friends and relatives; these costs exceed 20% of annual per capita household expenditure in 60% of Indian households with a patient with cancer.^9^ Estimates show that Indian citizens spent 6.74 billion US dollars in 2012 as a result of cancer deaths.^11^

Cancer mortality in India has doubled from 1990 to 2016.^12^ India’s cancer incidence is estimated at 1.15 million new patients in 2018 and is predicted to almost double as a result of demographic changes alone by 2040 (Table 1).^13^ Public cancer facilities in India are woefully inadequate, and there is large presence of private cancer care facilities.^17^ Some have exploited this situation by selling vulnerable patients unproven therapies to prevent, cure, or control cancer.^18,19^ As a result of the great increase in cancer, all public cancer treatment facilities are overcrowded and teeming with patients, resulting in India’s cancer problem being called an epidemic or a tsunami.^20-24^ The reasons for the increase in cancer are enigmatic, and the popular media and lay public regularly blame erosion of traditional Indian culture and Westernization.^21^ Historically, a similar situation had occurred in England in the latter half of the 19th century, which led King and Newsholme^25^ to publish a paper titled, “On the Alleged Increase of Cancer” to explain the alarming increase in cancer deaths. Much discussion and debate followed this study, and in 1907, Bashford published an article titled, “Real and Apparent Differences in the Incidence of Cancer.”^26^ Similar alarms were raised in the United States and Canada.^27-29^

**TABLE 1:**
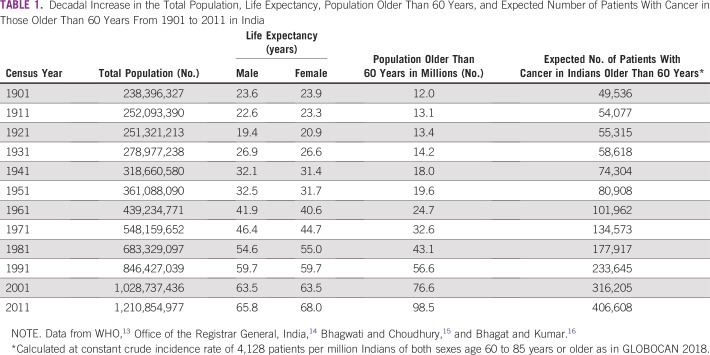
Decadal Increase in the Total Population, Life Expectancy, Population Older Than 60 Years, and Expected Number of Patients With Cancer in Those Older Than 60 Years From 1901 to 2011 in India

**CONTEXT**

**Key Objective**
The massive increase in cancer in India is enigmatic. A million new patients with cancer were diagnosed in 2018, overwhelming all the public cancer treatment facilities and making cancer treatments the leading cause of catastrophic out-of-pocket spending. We sought to trace the growing burden of cancer in India from antiquity.
**Knowledge Generated**
We found that although cancer has been recognized since the ancient Ayurvedic period, the routine diagnosis of cancer began in the 19th century after Western medicine gained acceptance throughout India. The incidence of cancer started to increase in the 20th century, when life expectancy began to increase in India. Cancer incidence rates in the Indian states vary widely, matching the states’ epidemiological transition levels in the 21st century. States with high epidemiological transition levels have lower premature mortality rates from infectious diseases, higher life expectancies, and higher incidence rates.
**Relevance**
The highly variable interstate increase of cancer incidence offers valuable lessons for India, its neighbors, and other developing countries for improving their cancer preparedness.

There is no comprehensive historical review explaining the growing burden of cancer in India. This review summarizes the history of cancer prevalence in India from antiquity and discusses measures taken, and not taken, to address India’s cancer burden over time. The historical facts regarding the increase in cancer can offer valuable insights and lessons to less developed Indian states, India’s neighbors, and other developing countries that are sure to face this problem in the future.^30^

This descriptive review used multiple search strategies to identify publications with facts and figures on cancer in India from the earliest possible time. We searched PubMed, PubMed Central, and Google Scholar databases; the British Library and the Wellcome Collection in London; the Tata Central Archives in Pune; and the Internet Archive digital library using the keywords cancer, malignant disease, neoplasms, and India. We searched the online publications and databases of many Government of India (GOI) departments and the databases of WHO and the International Agency for Research on Cancer for data on demography, epidemiology, and disease burden and cancer statistics from India (see Data Supplement for a detailed description of the data collection and its challenges).

## CANCER IN INDIA IN ANCIENT TIMES

A systematic review of 154 paleopathological studies has found evidence of cancer in early humans and hominins as far back as 1.8 million years.^31^ A paleoepidemiological study found similar age-adjusted malignant tumor rates in the skeletons found at an ancient Egyptian site and a southern German burial site compared with a recent control site.^32^

There are no paleo-oncology reports of cancer at the Indus Valley Civilization sites or elsewhere in India except for some benign osteomas.^33^ Higher prevalence of infections and trauma in the skeletal remains from Harappan burial sites resembles the current leading causes of death in the least developed regions of India.^34^ Inquiries were made to the researchers who had investigated the ancient Indus civilization sites (Harappan, Balathal, and Kalibangan) and Deccan Chalcolithic sites (Daimabad, Nevasa, and Inamgaon), and no additional noting of any malignant tumors in the skeletons excavated from these sites was found (G.R. Schug, N. Lovell, and V. Mushrif-Tripathy, personal communication, Dec 2018-Jan 2019). Fewer samples and poor preservation were some of the limitations of these studies.

There is no word equivalent to cancer in any of the texts from the Vedic ages.^22-24^ There are references to symptoms seen in advanced cancers and prayers and rituals seeking divine remedies in the *Atharva Veda*.^35^ India has two ancient medical systems, termed Ayurveda and Siddha, which have been used for more than 2,500 years.^35-39^ The Bower Manuscript, the earliest record documenting ancient medical systems of India, mentions diseases that would now be diagnosed as cancers.^40,41^ Cancer-like diseases (namely Arvuda, Granthi, and Gulma) are mentioned in the three main Ayurvedic classic texts, including the *Sushruta Samhita*, the *Charaka Samhita*, and the *Ashtanga Hridaya*.^42,43^ These texts mention the use of surgery and herbal medications for these diseases.^42,43^ The ancient medical classics of India have devoted little attention to cancer-like illnesses compared with more common diseases, suggesting a low prevalence of cancer in those times.^44^ The Siddha system of medicine, popular in ancient South India, mentions a cancer-like illness termed Puttru-Noi.^45,46^ Alchemy and toxic heavy metal preparations were used in the Siddha system and surgery was used in the Ayurveda system for managing several diseases. Autopsy, which was used to train in Ayurvedic surgery, declined during the Buddhist period starting around 400 BC. Some attribute the Buddhist concept of ahimsa, or law of nonviolence against man and animals, to have caused the decline in autopsies and surgeries, which halted the discovery of deep-seated cancers later on.^38^

Two other systems of medicine reached India during the early common era. The Sowa-Rigpa, better known as Tibetan medicine, describes a cancer-like disease called Dre-Nay.^47^ The Greco-Arabic system, called Unani-Tibbs medicine, describes a cancer-like disease called Sartan, meaning crab in Persian.^48^ The Ayurveda, Yoga, Unani, Siddha, and Homeopathy (AYUSH) and Tibetan systems are still used as the first treatment option or as complementary therapy by many patients in India.^49^ A search of the evidence-based research data portal of the Ministry of AYUSH of the GOI revealed little original work on cancer management.^50^ Of the 26,175 AYUSH publications containing the term cancer, only 15 were found to be grade A or grade B clinical trials. AYUSH treatments were used to complement non-AYUSH treatments in these trials. Collaborative studies are being planned between India and the United States to evaluate AYUSH treatments in cancer.^51^ The search did not reveal any original work on the burden of cancer in the AYUSH literature.

## CANCER IN INDIA AFTER THE ARRIVAL OF EUROPEANS

Western medicine reached India in the 16th century.^52,53^ Several European physicians and nonphysicians studied the plants, drugs, and formularies of India.^54-58^ In 1563, Gracia de Orta, a Portuguese physician who worked primarily in Goa, India, wrote *Coloquios dos Simples e Drogas da India*, documenting various medicinal plants of India, some of which were used on patients with cancer.^54,55^ The Dutch administrator of Cochin (now known as Kochi), Hendrik Van Rheede, cataloged the plants of Malabar (Kerala) from 1678 to 1693 and published the *Hortus Indicus Malabaricus* with help from native physicians.^55,56^ This book also contained references to the use of local plants in the treatment of cancer. The presence of cancer in India started to appear sporadically in some of the medical writings from the 17th century.^58^ However, there were no reports on the probable prevalence of cancer in India until the end of the 18th century. Until the early part of the 20th century, the life expectancy of Indians was low as a result of major famines and epidemics of infectious diseases (Data Supplement).^59,60^ The creation of the Indian Medical Service (IMS), staffed by European trained doctors, was a major milestone for India.^61^ The IMS surgeons began to diagnose cancers, and early IMS publications, including “Sketches of Most Prevalent Diseases of India”^62^ and “A Catalogue of Indian Medicinal Plants and Drugs,”^63^ made passing references to cancer.

Increasing demand for Western physicians led to the opening of medical colleges in the larger cities of colonial India, beginning with Calcutta (now known as Kolkata) Medical College in 1835.^64^ Cancer was being diagnosed in all parts of India, and clinical audits began to include cancers among native Indians.^65^ In 1840, F.H. Brett from Calcutta published *A Practical Essay on Some of the Principle Surgical Diseases of India*, which states that malignant diseases were prevalent in eastern India.^66^ In 1856, C. Morehead from Grant Medical College in Bombay (now known as Mumbai) published a book on the diseases of India and documented cancer cases from western India.^67^ Scientific work on the prevalence of cancer in India had begun.

In 1866, W.J. Elmslie was the first to publish a series of 30 patients with epithelioma, including the unique cancer associated with the use of a kangri pot, among 5,080 patients from Srinagar in Kashmir.^68^ The kangri pot is an indigenous device holding smoldering coal that is kept between the legs or in contact with the abdomen to warm a person through cold winters. From 1880 to 1910, there were more than a dozen case series and audits published on cancer in India. In 1880, Tomes^69^ reported five patients with cancer seen over 7 weeks in a dispensary in rural Bengal. The Kashmir Mission Hospital reported 2,020 cancers from their pathology reports between 1890 and 1899.^70^ Sixteen cancers among 450 autopsies were found between August of 1898 and June of 1900 at Calcutta Medical College.^71^ Among the autopsies done at Madras (now called Chennai) General Hospital from 1892 to 1901, 1,370 cancers were found.^72^ Surgical audits of a large number of patients were reported from Punjab (northwestern) and Travancore (now called Tiruvananthapuram) (southern). India had many patients with cancer (Data Supplement).^73,74^ Patients diagnosed with cancer were predominantly male, because women rarely used Western medical facilities given cultural norms. The importance of older age in the development of cancer was recognized, and the difficulties in obtaining the real age of native Indians were stressed in the clinical manual for India in 1897.^75^

In India, the initial reports had an excess of superficial cancers that were easy to diagnose, and several unique types of cancer were also described. These included kangri cancer (caused by the kangri pot), cheek cancer (caused by a betel nut–tobacco mix kept in the buccal sulcus), penile cancer (attributed to poor penile hygiene in uncircumcised men), dhoti cancer of the waist (caused by the dhoti, a loin cloth that is tied tightly around the waist), and scalp cancers (attributed to frequent tonsuring of the scalp).^68,76^ Chronic irritation of the epithelium or mucosa by thermal, physical, or chemical agents was hypothesized to cause these cancers. Most of these unique cancers have almost disappeared from India. Unfortunately, the habit of betel nut and tobacco chewing has increased all over India, and consequently, cheek and oral cancers are now among the top three cancers in most parts of India (Table 2).^24,77^

**TABLE 2:**
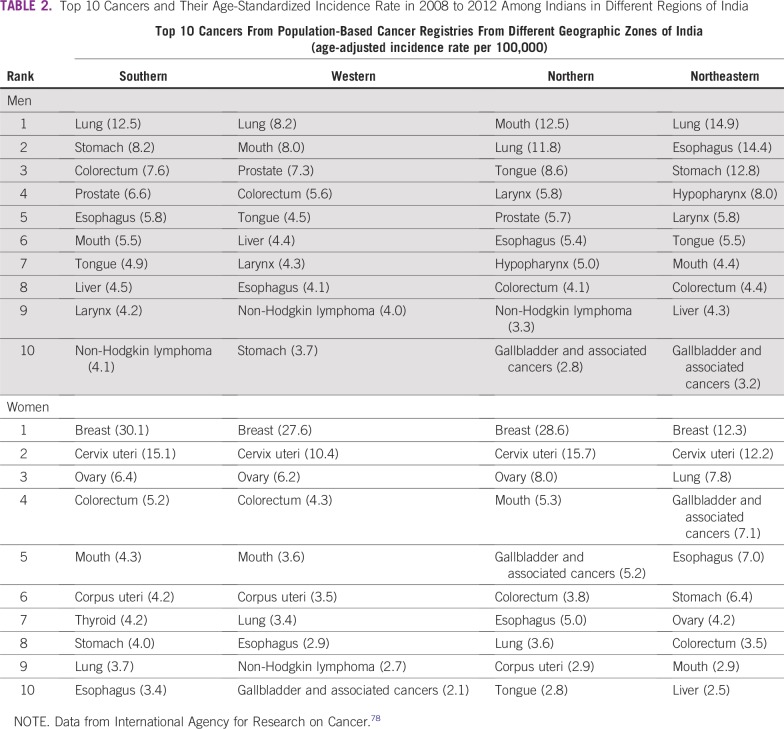
Top 10 Cancers and Their Age-Standardized Incidence Rate in 2008 to 2012 Among Indians in Different Regions of India

**TABLE 3:**
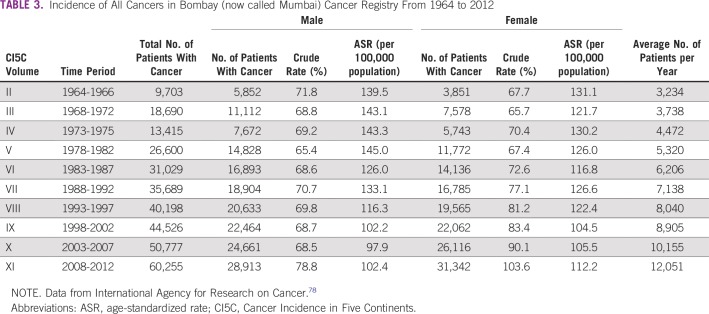
Incidence of All Cancers in Bombay (now called Mumbai) Cancer Registry From 1964 to 2012

These reports of cancer in natives of India fueled debates surrounding the role of geography, environmental factors, and race in the development of cancer.^71,79-81^ The prevailing idea that cancer was rare among the natives of India was discussed in British Parliament several times between 1902 and 1906.^82^ David Sutherland stated that “cancer is not a common disease in the Punjab” after reporting a 1.8% prevalence.^73(p90)^ Leonard Rogers, an experienced pathologist from Calcutta, stated that “malignant disease not only occurs in natives in India, but may be said to be common among them” based on his autopsy studies.^71(p280)^ Lazarus-Barlow from the Middlesex Hospital in London raised doubts regarding the validity of cancer diagnosis in India after comparing his data to the Punjab data.^79^ After inquiries from British Parliament, the Imperial Cancer Research Fund found 2,000 Indians with cancer in IMS hospitals from 1906 to 1908, concluding that cancer mortality in India “may not be markedly different from the English mortality [rate].”^81(p2)^ In 1908, Roger Williams wrote a book titled *The Natural History of Cancer*, in which he stated that “after careful study of all available sources of information, it appears to me clearly that malignant tumors are less prevalent, pro rata, in India than in Europe,” thus sustaining the debate.^83(p33)^ Leonard Rogers provided the best evidence supporting the increasing cancer burden in India with the help of a large autopsy study concluding that cancer is common in Indians when adjusted for age (Data Supplement).^84^

## CANCER IN 20TH-CENTURY INDIA

In the early 20th century, the real population-based incidence and mortality of cancer in India remained elusive as multiple barriers prevented data collection.^85^ These barriers included the nonavailability or the lack of access to medical facilities where cancer could be diagnosed, cultural habits preventing the use of medical facilities by native Indians, lack of knowledge and skills to diagnose cancer among native AYUSH doctors, lack of recording and notification of cancer diagnosis, lack of compulsory certification of deaths by medical doctors, omission of cancer diagnosis on death certificates, cover up of cancer diagnosis because of social stigma, and a lack of awareness among the native population regarding cancer and its causes and management. These limitations continue to exist in the less developed parts of India (Data Supplement).

Despite these barriers, the case for the growing cancer burden continued. In 1927, Megaw and Gupta^86^ conducted a nationwide survey of IMS doctors on the prevalence of disease seen in 202 district medical facilities in colonial India. Breast cancer was the most prevalent cancer, ranked as common in 98 institutions, followed by mouth cancer in 63 institutions, uterine cancer in 58 institutions, skin cancer in 35 institutions, and stomach cancer in nine institutions.^86^ Nath and Grewal^87-89^ conducted a landmark study across India funded by the Indian Research Fund Association. They collected autopsy data, pathology data, and clinical data from all large teaching medical institutions in India from 1917 to 1932.^87-89^ Cancer was found to be an important cause of adult death in all parts of India. Their nationwide data showed that no community or region of India was free from cancer (Data Supplement). They reported aging to be the most important determinant of cancer in Indians. Nath went on to contribute to various health care policy committees of preindependent India.^90,91^ The Indian Medical Review of 1938 stated that “[cancer] affords significant evidence as to this position being not insignificant” and advised IMS doctors to take the threat of cancer seriously.^90(p224)^

John Spies, an American cancer surgeon and brachytherapist, was consulted before building India’s first comprehensive cancer hospital, the Tata Memorial Hospital (TMH) in Bombay.^92^ Spies researched the incidence of cancer in Bombay and estimated that there were approximately 3,000 people in Bombay afflicted with cancer annually.^92^ The 1933 to 1934 annual report of the King Edward Memorial Hospital in Bombay also raised the issue of the need for cancer treatment facilities in Bombay.^93^ The TMH was inaugurated in 1941.^94^

The Health Survey and Development Committee of India, commonly called the Bhore Committee, was established to recommend the health needs of India immediately before independence. This committee published a report in 1946 that concluded that cancer prevalence in India was “not insignificant” and recommended several measures to improve cancer care in India (Data Supplement).^91(p116)^

## CANCER IN POSTCOLONIAL INDIA

Soon after India’s independence, the GOI committee for Indian systems of medicine (AYUSH) did not include cancer in its report.^95^ However, in 1948, an upgrading committee of the GOI visited the Pathology Department of TMH and recommended it be developed into the Indian Cancer Research Center with V.R. Khanolkar as its director.^96^ The Indian Cancer Research Center was later renamed the Cancer Research Institute in 1952.^76^ Khanolkar published several papers on the prevalence and etiology of cancer in India.^97-99^ He estimated cancer mortality in India to be 200,000 per year using hospital-based data from TMH.^98^ From 1950 to 1954, Khanolkar was the president of the International Cancer Research Commission. In his presidential address to the 1950 congress, he stated that “the experience of trained observers in modern medical institutions in India as far apart as Madras, Calcutta, Lahore and Bombay suggest that the incidence is much the same in Eastern countries as in Western Europe and North America. . . . It is found that the mean annual mortality from cancer in Bombay city per 100,000 living persons arranged by age groups approximates that of New York city [sic] and Zurich, if the suggested corrections are made.”^98(p883)^

In 1959, the second Health Survey and Planning Committee found India’s cancer care infrastructure to be “entirely inadequate.”^100^ They recommended that “each State should have a full-fledged hospital equipped with modern facilities for the surgery and radio-therapy of cancer cases” (Data Supplement).^100(p105)^ By 1960, only two more comprehensive cancer institutes were created in India, including the Chittaranjan National Cancer Institute in Calcutta in 1946 and the Cancer Institute in Madras in 1952.^101^

Mitra and Das Gupta carried out the first study to estimate India’s cancer burden using population-based data.^102^ Using the death registration records of the Calcutta Corporation, they found cancer to be the cause of 2.35% and 2.8% of deaths in 1954 and 1955, respectively.^102^ They estimated India’s national prevalence of cancer to be approximately 600,000 patients based on life expectancy, duration of survival after cancer diagnosis, and the size of India’s population. They reported a 10% higher mortality in young males, five times the prevalence of genital cancers in females, and an excess of gallbladder cancer compared with Americans.^102^

A nationwide audit of cancer among 1.03 million railway employees between age 18 and 55 years and their dependent family members from 1960 to 1964 was reported.^103^ This study found unique regional variations in the sites of several cancers, including buccal cavity cancers in the northern zone, stomach cancers in the southern zone, and biliary cancers in the northern and eastern zones.^103^ Population-based incidence, mortality, and prevalence for cancer in India remained speculative in 1964. Even in 1990, India’s facilities for the diagnosis and treatment of cancer were far behind recommendations.^104^

## POPULATION-BASED CANCER REGISTRIES IN INDIA

The first population-based cancer registry (PBCR) in India was created in Bombay in 1963 by Darab Jussawalla with funding from the Indian Cancer Society and the National Cancer Institute of the United States.^104^ This PBCR provided reliable population-based data on the cancer incidence in Bombay.^104-106^ Cancer incidence trends over half a century show the effect of demographic change on cancer burden (Table 2 and Fig 2). The incidence of cancer in Bombay has increased four-fold, even when the age-standardized incidence rates declined slightly (Table 2).^78^ More evidence that the demographical transition is the major determinant of the cancer burden can be found by comparing cancer incidence in the Parsees, a wealthy long-lived ethnic group, against that of other communities in Bombay (Data Supplement).^108^ India’s National Cancer Registry Program began in 1982, and several urban and some rural PBCRs have been added gradually.^109^ However, India’s most populous and least developed states, labeled as the Empowered Action Group (EAG) states, continue to lack PBCRs and accurate population-based data. The most recent estimates of India’s cancer burden reported in the GLOBOCAN 2018 database were created by extrapolating data from several regional PBCRs, with limited rural and no EAG state representation.^4,5^

**FIG 1:**
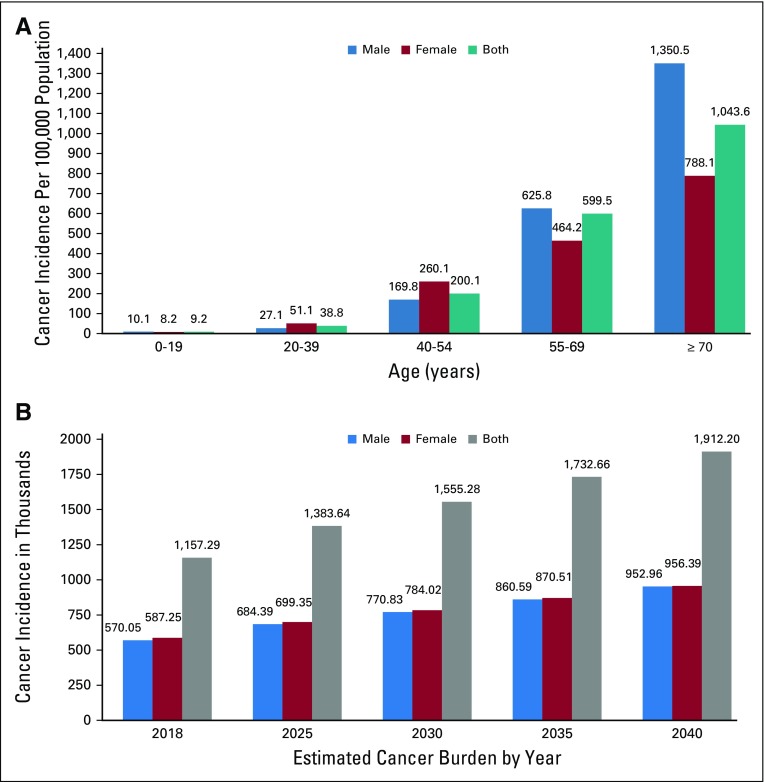
Cancer incidence in Indian men and women in 2018. (A) Incidence rates are per 100,000 population grouped by age and sex. (B) Projected cancer burden by sex from 2018 to 2040. Data from WHO.^13^

**FIG 2:**
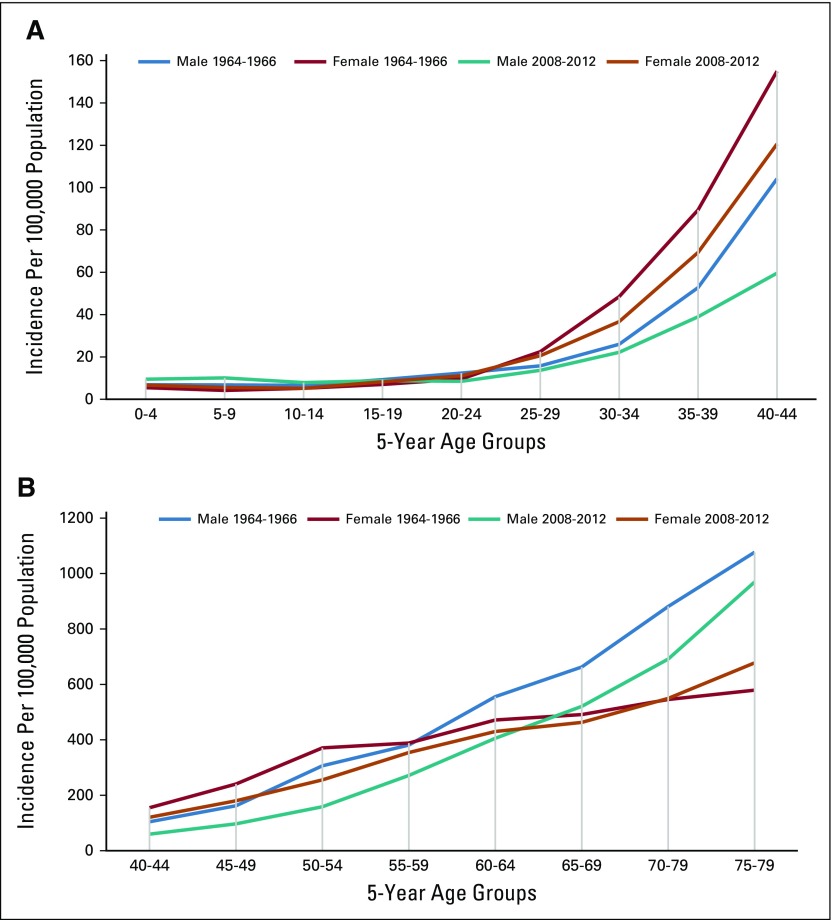
Age-specific incidence rates for all cancers per 100,000 men and women in Mumbai in 1964 to 1966 and 2008 to 2012. (A) Children and young adults. (B) Older adults. Data from WHO.^13^

## CANCER IN INDIA: LESSONS FOR DEVELOPING COUNTRIES

**FIG 3:**
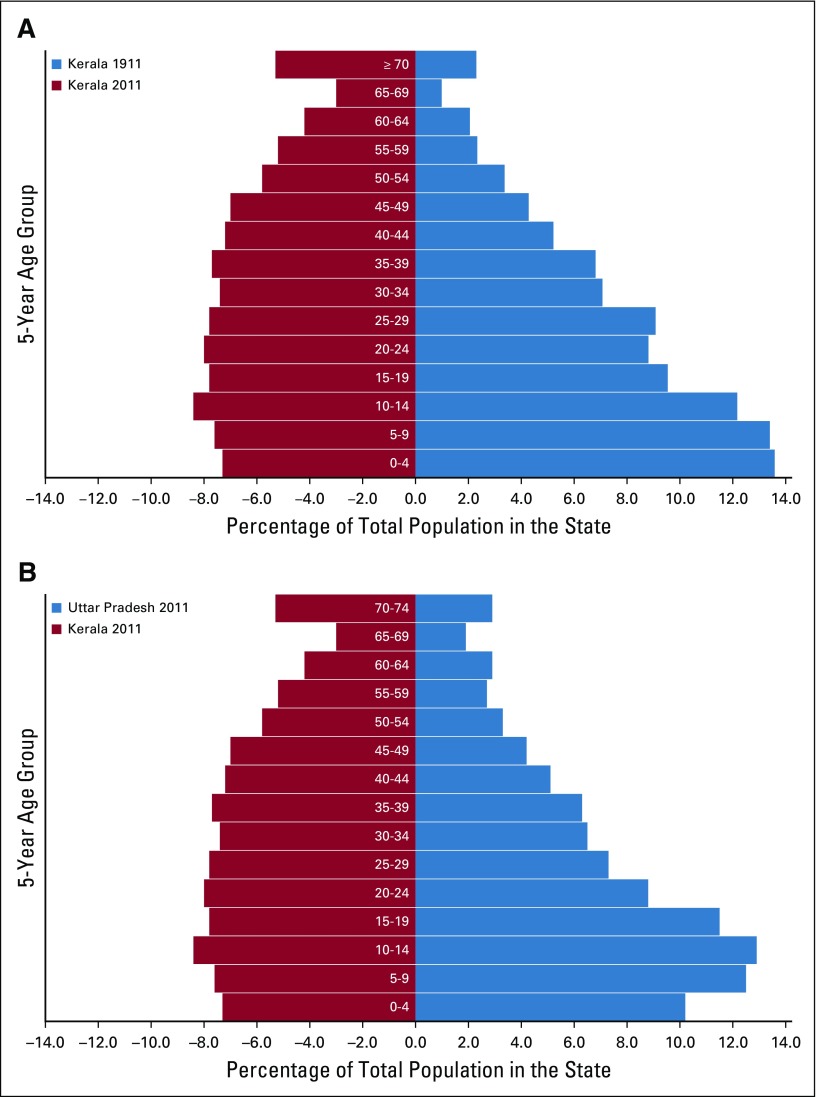
Epidemiologic transition in two states in India. (A) Population pyramid of Kerala state in 1911 and 2011. (B) Population pyramid of Kerala state in 2011 compared with Uttar Pradesh state in 2011. Data from WHO^13^ and Aiyar.^107^

**FIG 4:**
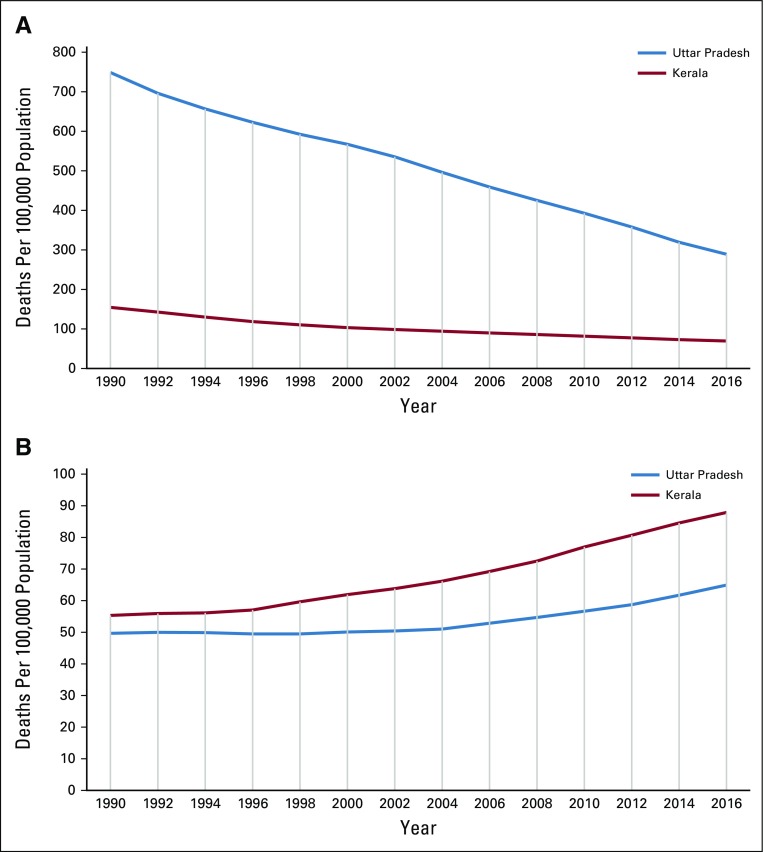
Time trends of death rates per 100,000 persons in Uttar Pradesh State and Kerala State over 26 years. (A) Deaths as a result of communicable, maternal, and neonatal diseases. (B) Deaths as a result of neoplasms. Data from Indian Council of Medical Research, Public Health Foundation of India, Institute for Health Metrics and Evaluation.^111^

**FIG 5:**
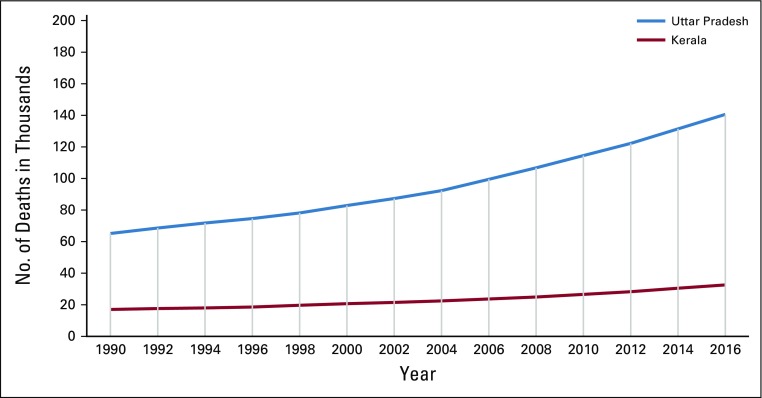
Time trends for the growing number of cancer deaths (in thousands) in Uttar Pradesh State and Kerala State over 26 years. Data from Indian Council of Medical Research, Public Health Foundation of India, Institute for Health Metrics and Evaluation.^111^

India has been described as “nations within a nation” because of the wide differences in the epidemiological transition levels (ETLs) among its states.^110^ The fastest epidemiological transition happened in the southern state of Kerala, whereas the most populous EAG state of Uttar Pradesh remained in the lowest ETL group (Fig 3).^110,111^ Comparison of the demographic and social variables, available health care facilities, and leading causes of mortality in these two states is revealing (Table 4 and Figs 3 to 5).^110-112^ The time trends for deaths from communicable diseases and neoplasms in these two states provide valuable insights for countries transiting from low ETL to high ETL.^111^ The types of cancers in India are also undergoing a transition, similar to a report from Japan five decades ago.^114^ There has been an observed decline of cancers in India caused by infections, such as cervical, stomach, and penile cancer, and an increase in cancers associated with lifestyle and aging, such as breast, colorectal, and prostate cancers (Tables 2 and 4). Cancer transitions can influence the requirements for site-specialized cancer surgeons, and countries following similar trajectories should expect similar challenges.

**TABLE 4:**
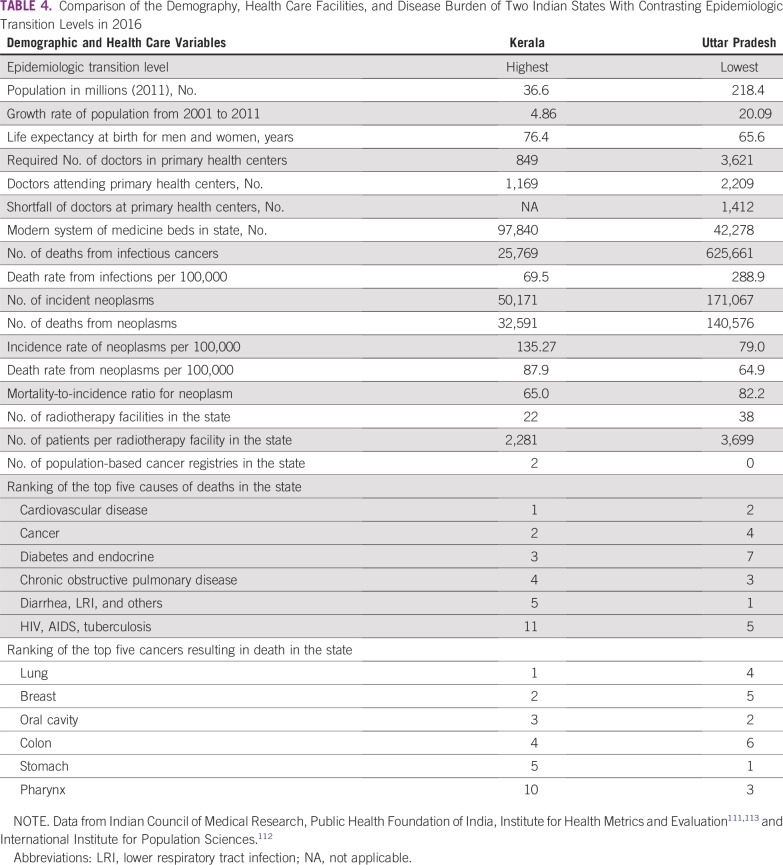
Comparison of the Demography, Health Care Facilities, and Disease Burden of Two Indian States With Contrasting Epidemiologic Transition Levels in 2016

The association of betel nut and tobacco chewing with cancer and warnings to stop the spread of this dangerous habit in India were published a century ago.^74^ This habit has remained unchecked and has spread all over India, and it is now estimated to cause one fifth of all cancers in India (Table 2).^77^ All states and countries must enforce a strong tobacco control policy to prevent cancer. Indian data also show that the populous low-ETL states will start to transition faster in the coming decades, and their cancer burdens will similarly increase. Paradoxically, these states have already accumulated a huge gap in cancer care, and even more effort will be needed in the future to close these gaps (Data Supplement). Inadequate cancer care facilities will cause more delays in diagnosis and treatment. The shortage will also force many to travel long distances to seek better cancer care, thereby increasing costs and OOPE. Developing countries must build their cancer care facilities with adequate manpower within easy geographical reach of their patients so as to reduce delays, reduce overhead costs, and facilitate complete treatments.

Government committees in India had identified the growing burden of cancer and proposed management plans nearly eight decades ago, but they have been unable to implement these recommendations. Currently, more than one million new patients need cancer treatment in India per year, but the cancer treatment facilities, manpower, and training have lagged behind (Data Supplement). The widening gap has facilitated rapid growth of for-profit private cancer care and onco-tourism, leading to increasing costs; more OOPE; more treatment dropouts; and avoidable physical, mental, and financial suffering for all stakeholders in India.

## THE FUTURE OF CANCER IN INDIA

India’s epidemiologic transition was triggered by large reductions in premature deaths from infections and associated diseases and increased life expectancy (Table 5 and Fig 4). Thus, all Indian states are experiencing an increase in cancer and other noncommunicable diseases.^111^ Cancer diagnoses are still missed in the least developed and rural parts of India as a result of the lack of adequate and easily accessible cancer care facilities. In 1993, an autopsy study from India’s premier postgraduate medical institute revealed that 25.8% of cancers were incorrectly diagnosed.^115^ Data quality in Indian urban cancer registries is still prone to errors.^116^ The 2018 quality report from Cancer Incidence in Five Continents indicates that 23% of cancers in rural Assam were unclassifiable.^117^ The increasing availability of minimally invasive diagnostic technologies, including image-guided needle aspiration cytology and immunohistochemistry, will further increase cancer diagnosis in India.^118^ The introduction of computed tomography scanning in Mumbai in the mid-1980s was immediately followed by an increase in the incidence of brain tumors, which stabilized later.^119^ Reduction of cardiovascular disease mortality is correlated with increased cancer mortality in many developed countries.^120^ Further reduction in cardiovascular disease mortality, which is presently three times higher than cancer in India, will increase the cancer burden further. Cancer screening, which is being considered by the GOI, is known to increase incidence while reducing mortality.^121,122^ All these factors will lead to further increases in India’s future cancer burden.

**TABLE 5:**
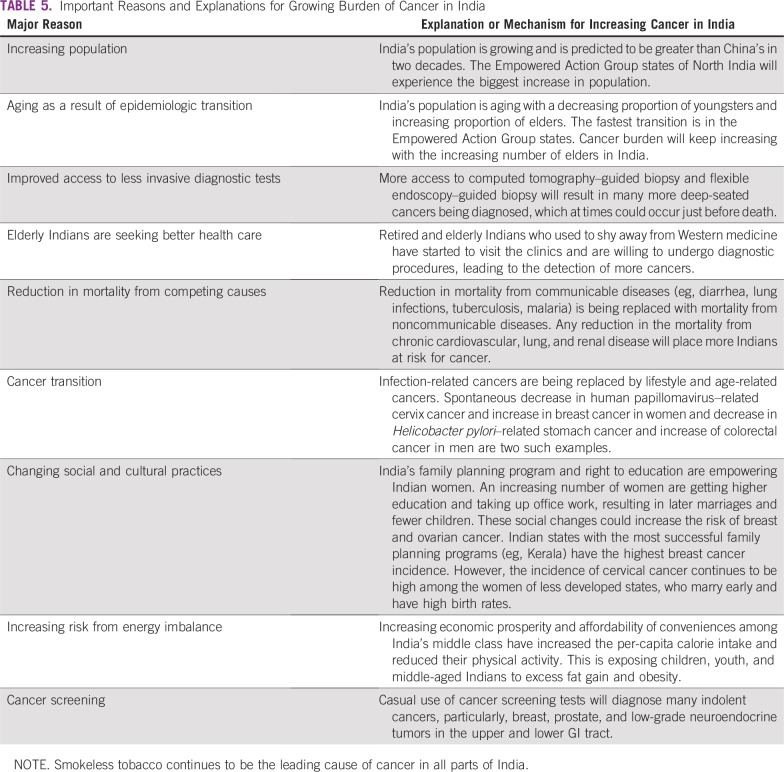
Important Reasons and Explanations for Growing Burden of Cancer in India

## CONCLUSION

Cancer-like illness has been documented in the Indian subcontinent since antiquity. The actual diagnosis of cancer began in the 19th century, and the cancer burden started to increase in the 20th century. Several pioneers had sounded warnings on the growing burden of cancer in India throughout the past century, but their warnings were heeded late. Most of the increase of patients with cancer in India is attributable to its epidemiologic transition and the improvement and increased use of cancer diagnostics in India. India’s cancer burden will continue to increase as a result of the continuing epidemiological transition. Maximum increases will occur in the most populous and least developed EAG states, where the current cancer treatment facilities are grossly inadequate.^123^ This review can provide many developing countries with valuable insights and important lessons based on India’s experience to enable them to avert similar crises in their countries.
